# Network-driven plasma proteomics expose molecular changes in the Alzheimer’s brain

**DOI:** 10.1186/s13024-016-0095-2

**Published:** 2016-04-26

**Authors:** Philipp A. Jaeger, Kurt M. Lucin, Markus Britschgi, Badri Vardarajan, Ruo-Pan Huang, Elizabeth D. Kirby, Rachelle Abbey, Bradley F. Boeve, Adam L. Boxer, Lindsay A. Farrer, NiCole Finch, Neill R. Graff-Radford, Elizabeth Head, Matan Hoffree, Ruochun Huang, Hudson Johns, Anna Karydas, David S. Knopman, Andrey Loboda, Eliezer Masliah, Ramya Narasimhan, Ronald C. Petersen, Alexei Podtelezhnikov, Suraj Pradhan, Rosa Rademakers, Chung-Huan Sun, Steven G. Younkin, Bruce L. Miller, Trey Ideker, Tony Wyss-Coray

**Affiliations:** Department of Neurology and Neurological Sciences, Stanford University School of Medicine, Stanford, CA USA; Institute of Chemistry and Biochemistry, Free University Berlin, Berlin, Germany; Departments of Bioengineering and Medicine, University of California San Diego, La Jolla, CA USA; Department of Medicine (Biomedical Genetics), Boston University Schools of Medicine, Boston, MA USA; RayBiotech, Guangzhou, China; RayBiotech, Norcrosse, GA USA; Department of Neurology, Mayo Clinic, Rochester, MN USA; Department of Neurology, University of California San Francisco, San Francisco, CA USA; Departments of Neurology, Ophthalmology, Genetics and Genomics, Epidemiology, and Biostatistics, Boston University Schools of Medicine and Public Health, Boston, MA USA; Department of Neuroscience, Mayo Clinic, Jacksonville, FL USA; Department of Neurology, Mayo Clinic, Jacksonville, FL USA; Departments of Pharmacology and Nutritional Sciences and Sanders-Brown Center on Aging, University of Kentucky, Lexington, KY USA; Department of Computer Science and Engineering, University of California San Diego, La Jolla, CA USA; Genetics and Pharmacogenomics, Merck Research Laboratories, West Point, PA USA; Department of Pathology, University of California San Diego, La Jolla, CA USA; Center for Tissue Regeneration, Repair and Restoration, VA Palo Alto Health Care System, Palo Alto, CA USA; Present address: Biology Department, Eastern Connecticut State University, Willimantic, CT USA; Present address: Roche Pharma Research and Early Development, NORD DTA, Roche Innovation, Center Basel, Basel, Switzerland

## Abstract

**Background:**

Biological pathways that significantly contribute to sporadic Alzheimer’s disease are largely unknown and cannot be observed directly. Cognitive symptoms appear only decades after the molecular disease onset, further complicating analyses. As a consequence, molecular research is often restricted to late-stage post-mortem studies of brain tissue. However, the disease process is expected to trigger numerous cellular signaling pathways and modulate the local and systemic environment, and resulting changes in secreted signaling molecules carry information about otherwise inaccessible pathological processes.

**Results:**

To access this information we probed relative levels of close to 600 secreted signaling proteins from patients’ blood samples using antibody microarrays and mapped disease-specific molecular networks. Using these networks as seeds we then employed independent genome and transcriptome data sets to corroborate potential pathogenic pathways.

**Conclusions:**

We identified Growth-Differentiation Factor (GDF) signaling as a novel Alzheimer’s disease-relevant pathway supported by in vivo and in vitro follow-up experiments, demonstrating the existence of a highly informative link between cellular pathology and changes in circulatory signaling proteins.

**Electronic supplementary material:**

The online version of this article (doi:10.1186/s13024-016-0095-2) contains supplementary material, which is available to authorized users.

## Background

Plasma proteins provide a sampling of biological processes throughout the organism and have been applied to diagnose or monitor human disease. However, in neurodegenerative disorders it has so far been more difficult to use unbiased large-scale proteomic approaches to discover blood-based biomarkers for diagnostics [1–3]. While individual patient samples might be insufficient for reliable classification tasks based on plasma proteins alone, patient populations could instead be used to smoothen variability and identify underlying common changes linked to disease mechanisms. To achieve this, we propose a medium-scale proteomic strategy that concentrates on secreted signaling proteins involved in cellular communication. Changes in these signaling proteins may result from pathogenic processes or indicate cellular responses to disease. A screen focused on these proteins may not only reduce the proteome test space dramatically but also provide mechanistic insight [4]. Here, we examined whether this approach can robustly identify proteins and biological pathways linked to sporadic late-onset Alzheimer’s disease dementia (AD).

## Results

**Fig. 1:**
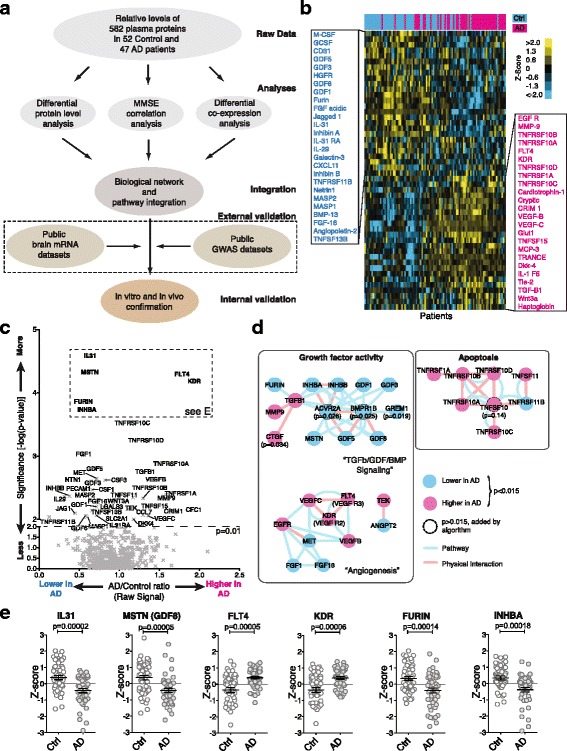
The circulatory AD signaling proteome reveals changes in cellular communication. **a** Overview of the experimental and analysis workflow. Plasma samples were collected at clinical centers, relative protein abundance was determined by antibody microarray and three types of analyses were performed: Protein level, MMSE correlation (cognitive performance), and protein co-secretion analysis. The analyses results were then integrated in a network and pathway enrichment framework and finally subjected to internal and external validation. **b** Heat map representation of the protein level analysis showing the top 50 most different proteins after unsupervised clustering (*q* < 0.05), separating samples into AD (pink, right) and controls (blue, left) and proteins into higher in control (blue, top) and higher in AD (pink, bottom). **c** Volcano-plot showing the distribution of all proteins and naming those significantly different between AD and control subjects (*p*_corr_ < 0.01). **d** A network representation of the most significantly changed proteins (*p*_corr_ < 0.015; un-connected proteins omitted) after integration with known pathway and physical interaction data reveals many densely connected hits in pathways related to TGFβ/GDF/BMP, angiogenesis, and apoptosis signaling. **e** Example scatter plots of the six top changed proteins (see dashed box in e, mean ± s.e.m; all *p*-values are corrected for multiple hypothesis testing)

To monitor the secreted signaling proteome in plasma, we manufactured glass-based microarrays with commercially available antibodies to measure the relative levels of close to 600 distinct secreted signaling proteins. Using these arrays, we obtained quantifiable results for 582 signaling proteins (Additional file 1: Figure S1A to D and Additional file 2) in archived blood plasma from 47 sporadic, cognitively impaired AD patients and 52 non-demented, closely age- and sex-matched controls obtained from two clinical centers (Additional file 1: Table S1). While these proteins do not encompass all secreted signaling proteins, they do provide a strong representation of all major signaling pathways and represent the largest dataset of this kind available today (Additional file 1: Figure S1A). Raw data were processed, normalized (Additional file 1: Figure S2), and then subjected to three parallel analyses, aimed at integrating both molecular and clinical data, followed by external and internal validation steps (Fig. 1a).

To identify signaling proteins with significantly changed plasma levels in AD we calculated corrected *p*-values for the quality controlled, centered, and normalized array data. Principal component analysis showed that our data were relatively free of obvious batch effects or confounding factors (Additional file 1: Figure S3). Clustering of the top 50 most different proteins illustrated clear differences between AD and control samples (Fig. 1b and c). Using the most significant proteins (FDR < 0.05, corresponding to *p*_corr_ < 0.015) as a starting point, we then queried known pathway or physical interaction databases to test the hypothesis that the signaling proteome could be mined to identify pathologically disturbed pathways (i.e., deregulated pathways should reveal themselves through changes in multiple receptors and/or ligands). This approach greatly reduces the chance of false-positive discoveries in contrast to following individually significant but unconnected leads. Using this methodology we identified highly interconnected clusters of receptors and ligands with growth factor activity (“TGFβ/GDF/BMP signaling” and “Angiogenesis”) or with activity linked to apoptosis (Fig. 1d). Reassuringly, the direction of changes was often coherent within each cluster/sub-cluster (Fig. 1d). Individual proteins can show highly significant differences between cohorts and, at the same time, exhibit large overlaps in the observed protein level ranges, highlighting the need for sufficient sample sizes and cohort stratification (Fig. 1e and Additional file 1: Figure S4).

To determine to what extent the observed changes in the AD signaling proteome are AD specific or the result of general neurodegeneration or other unrelated processes, we collected plasma samples from an additional 92 patients (Additional file 1: Table S2) suffering from semantic-variant primary progressive aphasia (svPPA), a sub-type of frontotemporal lobar degeneration (FTLD). SvPPA is almost always associated with Trans-activation response element (TAR) DNA-binding protein 43 (TDP-43)-aggregate pathology and appears to have weak genetic linkage [5–7]. This makes svPPA an ideal candidate to compare distinct neurological pathologies between two unrelated, sporadic, progressive dementias (svPPA *vs.* AD) *via* signaling proteome analysis [8]. The svPPA samples were prepared, handled, and analyzed in parallel to the AD samples to minimize experimental variations.

**Fig. 2:**
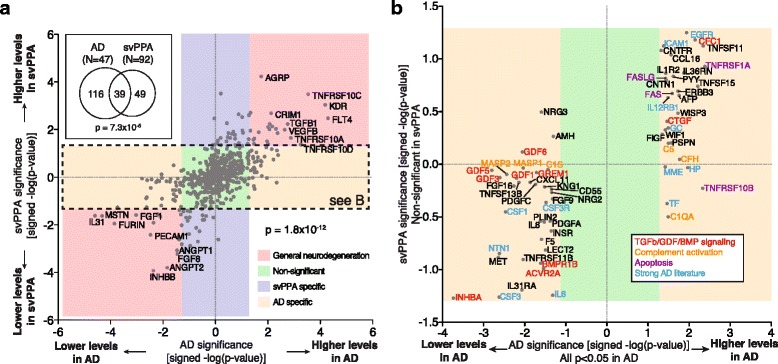
The plasma proteome contains disease specific information. To assess the specificity of the proteins identified in the expression level analysis, AD samples were compared to another, unrelated progressive dementia (svPPA = semantic-variant primary progressive aphasia). **a** Plotting signed, log-transformed *p*_corr_-values (more extreme = greater significance) of the AD *vs.* svPPA analysis show preserved directionality (binominal test) and can be used to categorize proteins into four distinct groups: “General neurodegeneration” (*p*_AD_ & *p*_svPPA_ < 0.05, same direction of changes in both diseases; red box); “Non-significant” (*p*_AD_ & *p*_svPPA_ > 0.05; green box), “svPPA specific” (*p*_AD_ > 0.05, *p*_svPPA_ < 0.05; purple box); “AD specific” (*p*_AD_ < 0.05, *p*_svPPA_ > 0.05; yellow box). Venn diagram showing the overlap of significantly changed proteins in AD or svPPA samples (top-left inset; threshold *p*_corr_ < 0.05; overlap significance by hypergeometric test). **b** Zooming into the “AD specific” box (see dashed box in a), many proteins can be identified as part of TGFβ/GDF/BMP, complement, or apoptosis signaling in addition to numerous proteins with strong supporting AD literature (manual curation)

We found 39 proteins with significant changes in both AD and svPPA (Fig. 2a, inset; *p* = 7.3×10^−5^ by hypergeometric test). Intriguingly, when we compared significance and direction of protein changes between the two pathologies, we found a perfect correlative trend of up/up or down/down amongst the 39 overlapping proteins (*p* < 1.8×10^−12^ by binominal test), indicating that a more general disease-profile does exist (Fig. 2a, red boxes). Additionally, we were able to identify proteins with svPPA-specific (Fig. 2a, purple box) and AD-specific proteome signatures (Fig. 2a and b, yellow box), respectively. A more detailed analysis of the svPPA findings is published elsewhere [9]. Manual literature curation of the AD-specific hits yielded numerous proteins involved in TGFβ/GDF/BMP signaling, complement activation, apoptosis, or with otherwise strong AD literature, suggesting that those pathways could play a role in AD (Fig. 2b). These findings indicate that a mixture of both disease-specific and nonspecific signaling profiles can be obtained from blood and that comparative proteomics can be applied to identify disease-specific changes.

**Fig. 3:**
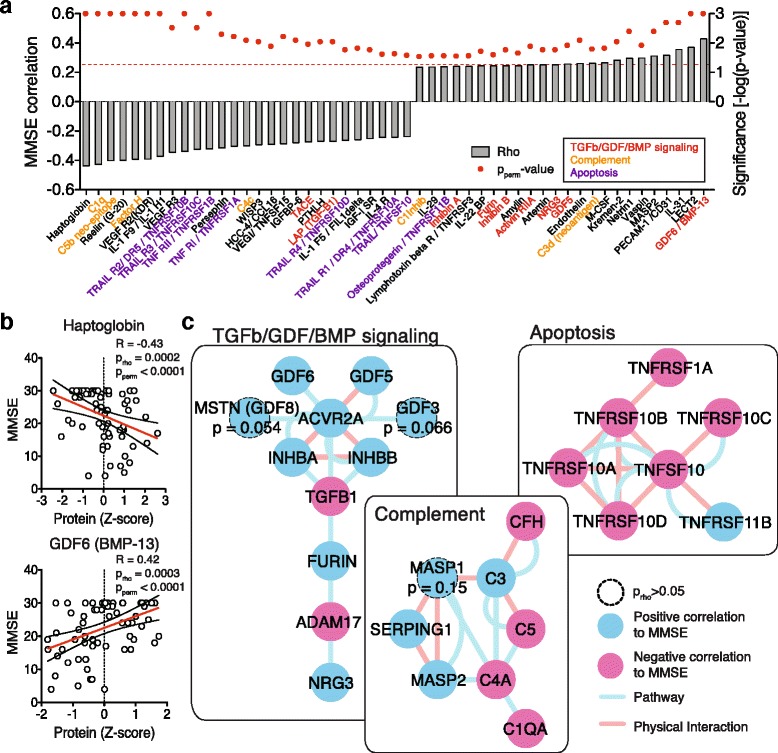
Correlation of cognitive function with the circulatory AD plasma proteome. **a** Proteins that exhibit significant correlation between cognitive function (evaluated by MMSE = Mini-mental state examination score) and protein levels ranked by correlation (cutoff *p*_rho_ < 0.05, Spearman rank correlation; *p*_perm_ based on 1000 MMSE-score permutations; dashed red line indicates *p*_perm_ = 0.05 threshold). Many proteins are part of TGFβ/GDF/BMP, complement, or apoptosis signaling. **b** Example scattergrams of the top positive and negative MMSE-correlated proteins (red line indicates regression with 95 % confidence intervals). **c** Network representation of significantly correlated proteins after integration with known pathway and physical interaction data reveals many densely connected hits in pathways related to TGFβ/GDF/BMP, complement, and apoptosis signaling

Changes in protein levels provide a binary view on AD (significantly changed or not). However, disease is a gradual process with patient’s cognition becoming increasingly more impaired. To explore the relationship between cognitive performance and relative plasma protein levels, we correlated the levels of 582 proteins with the Mini-mental state [10] examination (MMSE) scores of the respective patient (Fig. 3a and b, and Additional file 2; Spearman rank correlation and *p*-value [*p*_*rho*_] as well as explicit *p*-values through 1000-fold sample permutations [*p*_perm_]). Applying the same network exploration method as before, we identified again many proteins involved in TGFβ/GDF/BMP signaling and apoptosis to be positively or negatively correlated with cognitive performance (Fig. 3c), lending further support to our AD-specific findings above. While proteins involved in “Complement” activation were detected by the MMSE correlation analysis, they did not meet the cutoff criteria in the differential analysis, indicating that the complement driven effects are more subtle and gradual in nature (Fig. 3c). This finding highlights the fact that analyzing a continuous functional parameter such as MMSE can retrieve additional non-binary pathways of interest.

Testing for mean differences in plasma protein levels allows us to identify proteins with significantly different levels of expression between AD and controls. However, we hypothesized that additional insight could be gained from comparing changes in co-expression of signaling molecules as this might carry information on the pairwise relationship between the underlying signaling proteins and regulatory pathways, and on how this relationship is affected in disease [11].

**Fig. 4:**
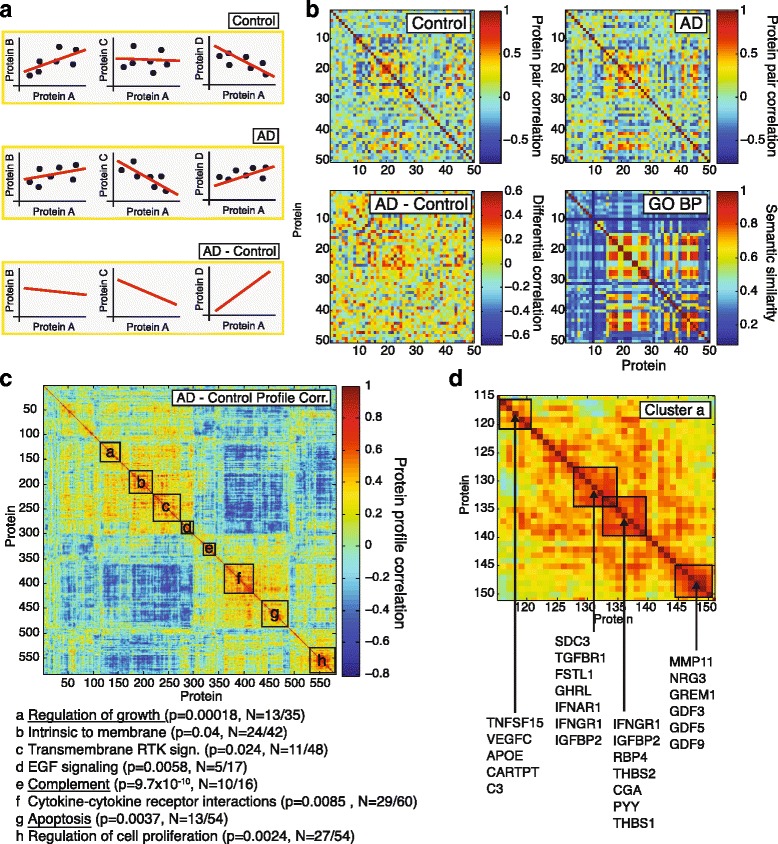
Protein co-expression analysis. **a** Schematic, hypothetical example of differential protein correlation: Proteins A to D exhibit a certain correlation pattern in control samples (top row) and a different pattern in AD samples (middle row). Subtracting the control correlations from the AD correlations yields the differential correlation “AD-Control” that captures the direction and magnitude of the correlation changes in disease (bottom row). **b** Zoomed-in correlation matrices for 50 proteins out of 582: Pairwise protein correlation in control samples (top left), AD samples (top right) and calculated differential correlation (bottom left; random subset of proteins in alphabetical order, Spearman rank correlation). “GO BP” represents the pairwise semantic similarity score of the protein pairs from ~0.1 (very different) to ~0.9 (very similar) in the “biological process” gene ontology as a measure for distance in the ontology tree and shared membership in biological processes. **c** Heat map of the differential profile correlations of all 582 proteins after unsupervised clustering with optimal leaf ordering. Positive correlations between two proteins indicate that these proteins change their correlations to many other proteins in a highly parallel fashion. Different clusters of proteins with similar profiles can be identified and are each significantly enriched for biological processes (boxes a-h, annotation below heat map; *p*-value based on a modified Fisher exact *p*-value; *N* = members with annotation/cluster size; three most significantly enriched clusters are underlined). **d** Cluster “a Regulation of growth” zoomed in with detailed sub-structure (same color scale as c)

To assess changes in co-expression, we calculated the Spearman correlation between each protein pair under healthy control and under AD conditions and then subtracted these correlations from each other, creating co-expression and differential co-expression networks respectively (Fig. 4a and b). It can be shown that these networks carry valuable biological information by comparing co-expression profiles and gene ontology (GO) biological process similarity between genes/proteins (Additional file 1: Figure S5). We found that protein pairs with high differential co-expression profile correlation (*R* > 0.35; Additional file 1: Figure S5) have significantly higher median GO semantic similarity scores than expected by chance, indicating that these protein pairs are functionally related.

Having established that meaningful biological information is contained within the co-expression profiles, we used hierarchical clustering [12] to arrange signaling proteins based on their co-expression profile correlations (Fig. 4c). Using DAVID [13, 14], we identified several clusters of proteins that were significantly enriched for a number of ontology terms (Fig. 4c). These protein clusters represent signaling molecules whose co-expression profiles change in a highly parallel fashion in AD, which in turn could indicate that the underlying regulatory pathways participate in AD pathogenesis. The most significantly enriched clusters were “Complement” (*p* = 9.7 × 10^−10^), “Regulation of growth” (*p* = 0.00018) and “Apoptosis” (*p* = 0.0037; all EASE score p-value based on 562 unique gene background). In this approach, the size (and number) of clusters identifiable is limited by the overall number of proteins probed: While it is apparent that cluster a-d and e-g could be grouped into larger “superclusters”, probing these superclusters for enrichment is not useful as they contain 25–45 % of all probed proteins (Fig. 4c).

Exploring the “Regulation of growth” cluster in more detail, we observed several sub-clusters that partially aligned with known biological relationships, often bridging regulatory pathways together (Fig. 4d). For example APOE deficient mice are prone to atherosclerosis, but C3 modulation of lipid metabolism can protect them [15], while VEGFC is a marker for advanced atherosclerosis and hypercholesterolemia in the same animals [16]. TGFβ and interferons (IFN) have antagonizing relationships in the control of inflammation [17], while interferons reduce ghrelin (GHRL) expression [18], and Follistatin-like 1 (FSTL1) is controlled by TGFβ [19]. We also noticed that numerous proteins were directly or indirectly associated with or regulated by TGFβ/GDF/BMP signaling (e.g., TGFBR1, FSTL1, THBS1, MMP11, GREM1, GDF3, GDF5, GDF9), making that pathway a lead candidate to test for its involvement in AD. These data suggest that co-expression analysis can be used to identify clusters of proteins and regulatory pathways that are linked through similar co-expression profiles, potentially indicating pathways that are affected by or affecting AD pathology simultaneously.

**Fig. 5:**
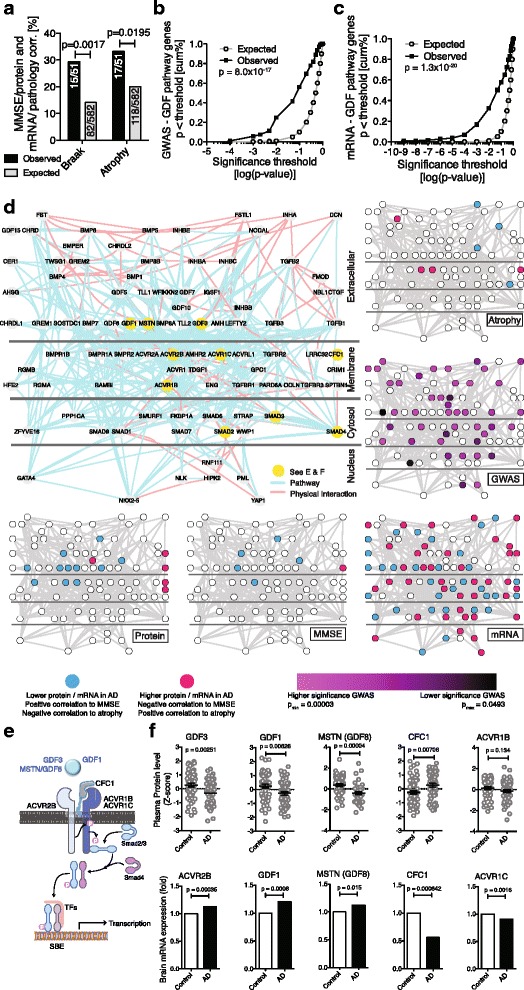
External Validation. To assess the biological validity of our findings top proteins were cross-referenced at the transcript or genomic level to external datasets containing AD brain mRNA transcriptome data or AD genome-wide association data, respectively. **a** Pre-frontal cortex transcripts in AD brain corresponding to proteins that correlated with MMSE in our study (see Fig. 3a) correlate strongly with Braak stage or brain atrophy (pre-frontal cortex) beyond what is expected by chance (chi^2^-test). **b** Based on meta-data from AD GWAS studies 114 genes which are part of “TGFβ/GDF/BMP signaling” exhibit higher than expected enrichment for significant SNPs (gene-wide *p*-value using VEGAS; cumulative curve comparison by Kolmogorov–Smirnov test). **c** Pre-fontal cortex transcripts for 120 genes of the TGFβ/GDF/BMP signaling pathway show many more significant transcript changes in AD than expected by chance (explicit p-value through sample permutation; cumulative curve comparison by Kolmogorov–Smirnov test). **d** Graphical representation of 92 proteins of TGFβ/GDF/BMP signaling pathway and integration with the findings from the various studies as indicated (only nodes with hits ≥ 1 are shown). Proteins are positioned relative to their location in the cells (extracellular, membrane bound, intracellular) and edges indicate physical or functional interactions (small border diagrams highlight proteins with associated significant changes). **e** Detailed pathway diagram for GDF-Activin receptor signaling. **f** Examples of protein (top row) and mRNA changes (bottom row) among the members of the GDF-Activin receptor signaling cascade (all corrected *p*-values)

Because protein quantification is notoriously difficult [1, 2, 20, 21] we examined the existing published literature for suitable non-proteomics data that could be used to validate our experimental approach. Recently, a large post-mortem study examined the mRNA expression levels in tissue from late-onset AD and control patients (cerebellum, pre-frontal cortex, and visual cortex) and provided data on the correlation between transcript levels and brain atrophy and Braak staging, which provides a measure of tangle pathology [22]. Using this dataset, we asked whether proteins that we found to be significantly correlated to cognitive performance (MMSE) also show a significant correlation between pre-frontal cortex transcript level and brain pathology (Braak stage and atrophy). And indeed, 29.4 % of the proteins (15/51) we had identified using MMSE as trait also have significant transcript-Braak stage correlations (*p* = 0.0017, Chi^2^-test, Fig. 5a), while 33.3 % (17/51) exhibited transcript-atrophy correlations (*p* = 0.0195, Chi^2^-test, Fig. 5a). This supports the notion that our experimental approach focusing on circulatory signaling proteins is able to enrich for proteins linked to AD brain pathology.

The results thus far from the differential protein level analysis, the correlations with cognitive function, and the co-expression network analysis consistently implicate the complement pathway, apoptosis, and a signaling network around the TGFβ superfamily in AD. We were particularly intrigued by this latter network, and its GDF family members, many of which had not been linked to AD. To further validate the significance of this finding we asked whether any of the proteins in this pathway – including intra- or extracellular ones not measured with the array – were additionally linked to AD at the genomic or transcript level. We used the Gene Ontology to identify a list of 114 genes linked to the TGFβ/GDF/BMP signaling pathway (Additional file 2) and queried two large AD data sets consisting of meta-data from 10 genome-wide association studies with a total of 8,309 AD cases and 7,366 cognitively normal elders [23] and AD brain transcriptome data from 181 AD and 125 controls [24] for significant SNPs or mRNA changes within the TGFβ/GDF/BMP pathway associated with AD.

We found significantly more SNPs in genes associated with TGFβ/GDF/BMP signaling than what would be expected by chance (*p* = 8.0×10^−17^, Kolmogorov-Smirnov test, Fig. 5b). Specifically, several genes that are involved in GDF3/Activin-receptor signaling had significant associations (GDF3, ACVR1B, SMAD3; see Additional file 2). Similarly, we found significantly more mRNAs with altered expression in AD brains associated with TGFβ/GDF/BMP signaling than what would be expected by chance (*p* = 1.3×10^−20^, Kolmogorov-Smirnov test, Fig. 5c and Additional file 2), including CFC1 (Cripto, a co-factor of GDF3), and Activin-receptor subunits 2B and 1C (ACVR2B/ACVR1C, GDF3 receptors). Taken together, multiple layers of experimental evidence suggest that changes at the genome, the transcriptome, and the proteome level in TGFβ/GDF/BMP signaling are associated with AD (Fig. 5d-f).

**Fig. 6:**
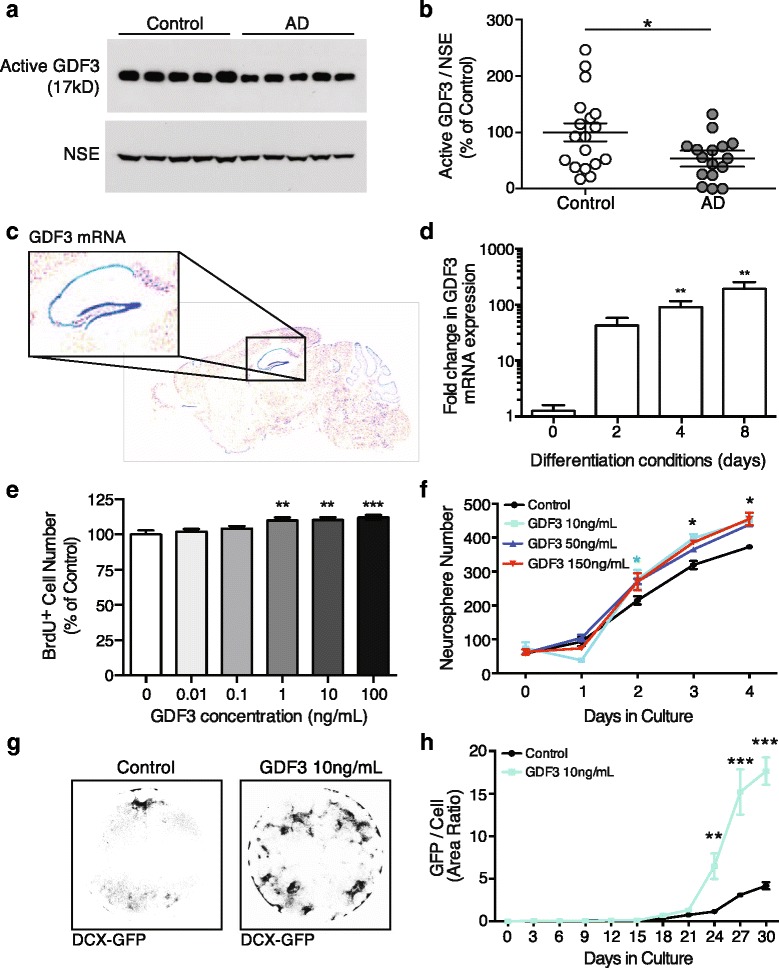
GDF3 regulates neurogenesis and is reduced in AD brains. **a** To test whether GDF3 levels are also reduced in AD brains, Human AD and control cortical grey matter regions were lysed and the detergent soluble protein fraction was probed by western blot. Levels of active GDF3 (**b**) were quantified relative to neuron-specific enolase (NSE). **c** To identify areas in the brain where GDF3 may have a functional role, we referred to The Allen Brain Atlas, which showed strong RNA expression in the mouse hippocampus (blue = high expression). **d** Using qPCR, GDF3 mRNA expression was detected in non-differentiated adult mouse NPCs and NPCs cultured in differentiating conditions. **e** To determine whether GDF3 affects stem cell function, adult mouse NPCs were provided recombinant mouse GDF3 and NPC proliferation was assessed using BrdU. **f** Recombinant mouse GDF3 was also provided to dissociated adult mouse neurospheres and the number of newly formed neurospheres was subsequently quantified. **g** To investigate whether GDF3 promotes neurogenesis, human-derived NTERA cells stably transfected with Dcx promoter-controlled eGFP were provided recombinant human GDF3. Shown are representative images of DCX-GFP fluorescence expression from an entire well of NTERA cells treated with GDF3 or control for 30 days. **h** DCX-GFP fluorescence area was quantified relative to the cellular area detected by brightfield microscopy. Results were compared by a one-way ANOVA with a Dunnett's post-test (**b**,**e**), an unpaired Student’s *t* test (**d**), or a two-way ANOVA with a Bonferroni post-test (**f**,**h**) and are representative of at least 2 independent experiments (b *n* = 16 –18 per group, d,e,f,h; *n* = 3 per group). Values are mean ± s.e.m.. *p < 0.05, **p < 0.01, ***p < 0.001 compared to respective control groups

Activin receptors and its ligands Inhibin A and B, GDF1, GDF3, GDF5, and the ligand binding proteins CFC1/cripto and gremlin were most prominently altered in AD throughout our study (Figs. 1d, 3c, 4d, 5f). While TGFβ and TGFβ-receptor signaling has been studied extensively in the context of AD and neuroinflammation [25, 26], and BMP9/GDF2 has been identified as a regulator of cholinergic neuronal development [27], Activin receptor signaling and GDFs have not been studied in AD. GDF3 represents a particularly intriguing candidate: it is highly expressed in the human brain [28] and mouse dentate gyrus (Fig. 6a), is part of the co-expression cluster a (Fig. 4c and d), exhibits significant SNP enrichment (Additional file 2), is positively correlated with cognitive performance (*p*_perm_ = 0.038), yet its effects on neurons or the brain are unknown.

Given that we observed reduced GDF3 plasma levels, we first investigated whether these changes may be reflective of GDF3 levels in human AD brains. To do this, we measured the processed, active form of GDF3 in tissue extracts of AD and age matched control patients and found a significant reduction in the cortex (*n* = 16–18 subjects per group, *p* = 0.02, Fig. 6b), but not in the cerebellum, an area unaffected by AD (*n* = 8 subjects per group, *p* = 0.15, Additional file 1: Figure S6A and B). To evaluate our svPPA *vs* AD comparison, which predicted that GDF3 reduction is AD-specific, we measured activated GDF3 in cortical extracts from svPPA patients and controls and found no difference in GDF3 levels (*n* = 5 subjects per group, *p* = 0.08, opposite trend to AD, Additional file 1: Figure S6 C and D). While these studies support our plasma proteomic findings, the source and functional significance of GDF3 within the brain remain unclear.

Because GDF3 is highly expressed in the mouse dentate gyrus (a neurogenic area; Fig. 6c) and previous studies show a role for GDF3 in regulating embryonic and cancer stem cell fate and differentiation [29–31] we asked whether GDF3 regulates adult neurogenesis. We first cultured primary adult mouse neural progenitor cells (NPCs) under non-differentiating and differentiating conditions and measured GDF3 mRNA. Inducing NPC differentiation into neurons and astrocytes caused a marked upregulation of GDF3 (Fig. 6d), suggesting that, unlike in embryonic stem cells [32], adult NPCs are not the main source of secreted GDF3 and adult hippocampal GDF3 likely derives from mature cell types [28]. To assess whether GDF3 affects NPC function, we exposed primary adult NPCs to increasing concentrations of recombinant mouse GDF3 and measured proliferation. Indeed, GDF3 increased NPC proliferation as measured by BrdU incorporation (Fig. 6e) and changes in neurosphere number (Fig. 6f). Given that GDF3 has been implicated in embryonic stem cell fate [29, 30] and is capable of differentiating PC12 cells [33], we next investigated whether GDF3 promotes neuronal differentiation. To evaluate neuronal differentiation we utilized NTERA cells stably transfected with Doublecortin (Dcx) promoter-controlled eGFP, as previously described [34]. NTERA cells cultured with GDF3 showed a prominent increase in Dcx expression compared to control cells (Fig. 6g and h), which was also confirmed by western blot (Additional file 1: Figure S6E and F). Furthermore, a recent study demonstrated the potential importance of developmental factors such as the Repressor element 1-silencing transcription factor (REST) in AD pathology [35]. Intriguingly, GDF3 and REST appear to be controlled by shared transcription factor binding [36], potentially hinting at common upstream disturbances. Taken together these findings suggest that GDF3 plays an important role in NPC proliferation and neuronal differentiation and that it’s levels are altered in AD.

## Discussion

This study uses a new approach to discover biological pathways associated with AD dementia by measuring hundreds of circulatory proteins involved in cellular communication. Combined with multiple levels of statistical and bioinformatics analyses and leveraging current pathway knowledge, we were able to build networks of proteins deregulated in AD. We provide evidence that this approach has considerable robustness for the discovery of proteins and pathways linked to AD and successfully validate it against existing datasets. We show that genomic and transcriptomic data can be used to corroborate suspected disease-specific pathways and identify GDF3 as a new regulator of adult neurogenesis. Given the important function adult hippocampal neurogenesis has in rodent behavior related to memory and learning, the recent discovery of significant hippocampal neurogenesis in the adult human brain even with advanced age [37], and previous implications of disturbed neurogenesis in AD [38, 39], changes in neurogenesis caused by abnormal GDF3 signaling might well have a role in the development or progression of AD.

Currently, our approach is limited only by the availability of high-quality, verified antibodies able to reliably bind signaling molecules in plasma. Future developments in the production of antibodies or antibody alternatives such as aptamteres [40, 41] will likely enable researchers to probe even broader sets of circulating antigens. However, with increasingly large numbers of analytes ever-closer attention has to be paid to the statistical methods involved in identifying interesting candidates. Independent sample cohorts that are probed using different experimental approaches should always be considered, similar to the approach we are taking in this study. Nevertheless, there are certain limitations to consider when pursuing the biological interpretation of high-throughput proteomic studies [42]: (1) Protein coexpression data is currently still fairly noisy, resulting in networks with noisy edges making cluster definition challenging. To deal with this problem we based most of our analysis on much more vetted pathway or protein-protein interaction data. (2) Most proteins act in transient and changing complexes and how to define these overlapping clusters is not trivial. (3) Similarly difficult is to avoid arbitrarily breaking large clusters into random smaller ones and to identify the “correct” number of clusters from *a priori* knowledge. (4) If possible, running replicates will reduce clustering error and using learning through training and test sets can similarly improve the validity of the results [43]. However, availability of precious sample material and cohort sizes often limits the ability to perform these steps. Encouragingly, these limitations are subject to very active ongoing research efforts and novel methodologies will likely enable us to avoid many of these pitfalls in the future.

Motivated by the idea to specifically monitor the cellular communication factors secreted into the bloodstream, we naturally had a strong representation of inflammatory and growth factor related proteins in our screen. In the complete human proteome, additional proteins and protein fragments have the potential to being secreted into the bloodstream and could provide mechanistic insight into further pathological processes active in AD, which we likely missed in our current screening format. Additional pathways or protein sets could easily be added to future versions of the screening platform as antibodies become available. Conversely, there may be other inflammatory and signaling-related pathologies that were left undiscovered due to limited sample numbers and increasing cohort sizes could ameliorate this limitation, although we estimate to have recovered 50–75 % of the true positive hits (Additional file 1: Figure S4). Finally, using gene ontology enrichment to identify classes of interesting genes inherently carries over the annotation and research bias present from GO [44]. This can be mediated somewhat by using more data-driven ontology approaches such as NeXO and CliXO [45, 46], although research bias cannot be overcome that way. We thus encourage the reader to not purely rely on the enrichment terms that we provide for the identified protein clusters, but to interpret the proteins of interest in the broader framework of biological knowledge.

Over the past decade, numerous studies [20, 21, 47–59] have been performed to identify biomarker sets in AD plasma samples (Additional file 1: Figure S1A), leading to the recognition of a number of biological pathways and proteins potentially affected in the disease [60]. Analyzing pathway enrichment in combined cohorts from multiple studies, Kiddle and colleagues [3] found the most significant enrichment in proteins linked to “Complement and coagulation cascades” (*p* = 9.78 × 10^−25^) and “Cytokine-cytokine receptor interaction” (*p* = 2.07 × 10^−18^), which is in good agreement with the pathways implicated in our screen (Figs. 3 and 4). Using a similar meta-analysis approach to identify individual proteins related to AD pathology, Chiam et al. report multi-cohort support for the involvement of Complement C3, Complement Factor H, and Plasma protease C1 inhibitor [60], all of which were identified in our screen as well (Fig. 3). For many of the other proteins and pathways implicated in this study (for example GDF), little comparable data exist, as they are not covered by the most widely used Rules Based Medicine (RBM) 190 analyte panel.

The approach presented here could be extended to study abnormal cellular processes in other CNS diseases or conditions where affected tissues are similarly difficult to access. Collectively, our data support the presence of an accessible and informative link between molecular (mRNA), cellular (Braak stages), structural (atrophy), and functional (MMSE) changes in the brain and circulatory signaling proteins. Exploiting this link, may aid in the design of novel therapeutic and early diagnostic strategies for sporadic AD.

## Conclusion

Neurodegenerative disorders are widespread, devastating, poorly understood, and largely untreatable with current knowledge. One difficulty lies in the fact that the brain is extraordinarily sensitive and cannot easily be examined on the cellular or molecular level in the diseased patient. Here, we instead created and analyzed a large set of blood protein data from Alzheimer’s disease and healthy control patients for changes in cellular communication factors, leading to the discovery and validation of altered GDF signaling in sporadic Alzheimer’s disease brain tissue. We achieve this by integrating multiple levels of ‘omics data, linking blood to brain tissue changes, thus opening up potentially new avenues for treatment and diagnosis.

## Methods

### Nomenclature

This is a proteomics study. To highlight this fact, we labeled hits in the first analyses (Figs. 1b and 2a) with protein names. Due to space restrictions in figure design, we labeled hits in all other figures with the HUPO *gene names* of the corresponding protein or mRNA products.

### Human plasma samples

All participants underwent thorough and standardized history and physical exams. For the antibody arrays, we used a total of 99 archived human plasma samples with ethylenediaminetetraacetate (EDTA) as anticoagulant collected at the University of California San Francisco (San Francisco, CA) and the Mayo Clinic (Rochester, MN and Jacksonville, FL; Additional file 1: Table S1). Plasma was produced by standard blood processing, then aliquots were frozen and stored in aliquots at −80 °C, avoiding freeze thaw cycles. Informed consent was obtained from human subjects according to the ethics committee guidelines at the respective clinical centers. All patients were clinically diagnosed with AD based on the 1984 NINCDS-ADRDA Alzheimer’s criteria with additional attention to the 2011 revisions [61] and (if possible) post-mortem tissue analysis (27 of the 47 AD cases). Details on the svPPA plasma samples are provided in Additional file 1: Table S2. A total of 92 svPPA patients from the University of California San Francisco or Mayo Clinic Jacksonville were identified whose clinical features conformed to revised consensus diagnostic criteria for svPPA [62]. Patient consent had been administered at the respective sample collection centers and research has been conducted according to the principles expressed in the Declaration of Helsinki. Analysis of de-identified samples was performed with research approval by the Stanford University institutional review board.

### Antibody-microarray production

Plasma protein levels were measured using antibody-based protein microarrays. We used a custom-expanded, commercially available microarray with modified antibody content (custom L-Series, RayBiotech Inc., Norcross, GA) containing 474 antibodies against chemokines and cytokines printed in triplicates by the company, plus 17 control antibodies. Additionally, we produced a custom-made in-house array that contained a separate set of 119 antibodies against secreted signaling factors printed in quadruplicates, plus 7 control antibodies. A total of 617 antibodies were measured. Subsequent quality control steps removed 11 antibodies with extremely low or no signal and control antibodies (see *Antibody-Microarray Data Preparation* below) yielding a total of 582 analyzed antibodies (Additional file 2 and Additional file 1: Figure S1 and S2C). Some antibodies target the same protein multiple times (such as precursor/full-length/truncated forms, 14 proteins and 32 antibodies total; see Additional file 2). The microarray production protocol was the following: antibodies of interest were selected based on their biological role as secreted signaling factors and the availability of ELISA-grade quality batches to ensure likely detection of the epitope in liquid solution. The arrays were printed onto SuperEpoxy glass slides (Arrayit, Sunnyvale, CA) using a custom-built robotic microarrayer fitted with sixteen SMP4B pins (Arrayit). After drying the slides overnight they were vacuum-sealed and stored at −20 °C until use.

### Plasma sample preparation and antibody-microarray incubation

The human plasma samples were thawed at room temperature and diluted 5-times in PBS without Ca^2+^/Mg^2+^ (pH 6.5) followed by 10,000 g centrifugation in a swing bucket centrifuge for 10 min at Room temperature. The lipid layer on top was carefully removed with the house vacuum. Without disturbing the platelet pellet 300 μl was carefully removed for dialysis (96 well Dispodialyzer/5 kDa, Harvard Apparatus, Holliston, MA) into PBS (pH 6.5) at 4 °C in multiple steps including a last over-night step to yield a maximally pure plasma protein fraction in an appropriate buffer for the biotinylation reaction. The dialyzed plasma was diluted again 6-times in PBS and recombinant Green fluorescent protein (GFP) was spiked into the samples as positive control at a final concentration of 1 μg/ml. The plasma proteins were N-terminally biotinylated (NHS-SulfoBiotin, Thermo Scientific, Rockford, IL), reaction was stopped with 0.1 M glycin final concentration and unbound biotin removed by multiple dialysis against PBS (pH 6.5 and last at pH 8). Then samples were diluted in 3 % casein in PBS (pH 7.4) and the individual samples were incubated on blocked antibody arrays over-night at 4 °C. Blocking was performed by incubating dried arrays in 4 °C precooled 3 % casein in PBS (pH 7.4) overnight on a shaker (30 rpm) at 4 °C. After multiple washing steps antibody-bound protein was detected using 0.5 μg/ml Alexa Fluor 555 conjugated streptavidin (Invitrogen) on a GenePix Pro 4000B scanner (Molecular Devices, Sunnyvale, CA, Additional file 1: Figure S1D). Samples for both AD and svPPA studies were processed in parallel in randomized order in one batch.

### Data processing and figure generation

Raw data from the array scanner were provided as images (.tif files) and spot intensities (Excel.xls files; Microsoft, Seattle, WA). Excel files were condensed into one file (tab-delimited.txt file) and non-analyzed data rows/columns were removed using RDBmerge (Ron de Bruin, www.rondebruin.nl). Unless otherwise stated, data processing and statistical testing were performed in Matlab R2012a (MathWorks, Natick, MT). Figures were generated directly in Matlab or data were transferred and plotted in Prism 5.0f (GraphPad Software, La Jolla, CA). Figures were then arranged for publishing using Illustrator CS5 and Photoshop CS5 (both Adobe, San Diego, CA).

### Antibody-microarray data preparation

To determine spot intensities, we calculated the mean pixel intensity per spot. To determine background intensities we calculated the median pixel intensity per background “doughnut” (Additional file 1: Figure S1D). Individual array spots were background subtracted locally (by subtracting the median background across spot replicates in each sample). Spots with a residual intensity less than 10 % above background were set to ‘ND’ (non-detectable). Antibodies with more than 55 % ‘ND’ values were excluded from the analysis (*N* = 11), yielding a total of 582 quantifiable antibodies (Additional file 2, for ‘ND’-count distribution see Additional file 1: Figure S2C). ‘ND’ values were then replaced with the greater of the half the minimum non-‘ND’ value per sample replicates, the half the minimum non-’ND’ value of that antibody across all samples (if the sample replicates were all ‘ND’), or 1. The spot data were Log2 transformed, replicate averaged, and iteratively (i = 50) row- and column-wise median centered (subtract the column-wise median from the values in each column/row of data, so that the mean or median value of each column/row is 0) and normalized (multiply all values in each column/row of data by a scale factor S so that the sum of the squares of the values in each column/row is constant across columns/rows) following a procedure described in the Cluster 3.0 manual [63]. Finally the data were Z-scored, leaving approximately normally distributed data for analysis with a mean of 0 and a standard deviation of 1 (Additional file 1: Figure S2D to F, 86 % of all antibodies have normal distributions based on one-sample Kolmogorov-Smirnov test).

### Principle component analysis

To assess the influence of potentially confounding factors such as plasma source, patient age, or patient gender, we performed a Principle Component Analysis in Matlab using the Z-scored data and the built-in *princomp* function.

### Differential protein level analysis

To identify proteins with significant changes in plasma levels (based on Z-score values) we calculated permutation-corrected *p*-values (*p*_corr_) for Control *vs.* AD for every protein (unpaired two-tailed *t*-test, 10,000 class label permutations) using the *mattest* Matlab function. To compute false-discovery rates, we adopted a direct approach to estimate *q*-values [64] using the *mafdr* Matlab function. Proteins with a significant difference between AD and Control samples and a *q* < 0.05 were considered having different plasma levels (a total of 50 proteins). An identical approach was used for the svPPA data.

### Network representation

To link the proteins with changed plasma levels to biological pathways, we mapped these proteins onto known protein networks using the Genemania-app [65] in Cytoscape 3.0.1 [www.cytoscape.org, [66]]. Pathway data came from NCI-Nature [67], Reactome [68–70], and [71]. Physical interaction data came from Biogrid Small Scale [72], IREF Interact, and IREF Small Scale [73]. We allowed for some nodes above the significance threshold to be added by the algorithm to connect cliques (dashed nodes, *p*-value indicted in figure). To test for enrichment in biological function, we queried Gene Ontology [74, 75], KEGG [76], and Panther [77] databases using DAVID [13] with the 564 unique genes representing the 582 proteins tested as background.

### Mini-mental State Exam (MMSE) correlation

MMSE scores were recorded at the time of plasma acquisition at the respective clinical centers. Scores were available for 44/47 AD patients and 26/52 Control patients (Additional file 2 and Additional file 1: Table S1). MMSE scores were correlated to Z-scored protein levels using Spearman’s rank correlation. Correlation significance was assessed using Spearman’s *p*-value (*p*_Rho_: significance that slope is not 0) and by computing an empirical *p*-value by permuting protein scores over MMSE scores 1,000-times (*p*_Perm_: Number of times that random MMSE-Protein data yields correlation greater than observed/1,000). Network representation was performed as described above.

### Differential co-expression analysis

Co-expression analysis was performed to identify proteins involved in AD pathogenesis that would inform us on more specific pathways than the broad ones implicated in the differential analysis above. Besides the mere difference in expression levels, proteins may differ in how they correlate with each other between disease and controls. We sought to discover protein networks that are changed between AD and Control by evaluating differential correlation matrices in an approach analogous to methods developed for analyzing genetic interaction profiles [78]. We thus created separate correlation matrices (Spearman’s rank correlation) in Matlab of all of the proteins measured for AD and Control signaling proteomes, respectively. Since cohort sizes were almost equal for AD and Controls we then calculated differential correlation profiles from these correlation matrices and used unsupervised clustering to identify 8 distinct clusters of proteins with highly similar differential correlation profiles (Fig. 4c, boxes). To demonstrate that differential co-expression data contains valuable biological information, we created semantic similarity scores for each protein pair [79]. Protein pairs with high differential co-expression profile correlation exhibited high semantic-similarity profile correlations as well (Additional file 1: Figure S5).

### Braak staging and atrophy correlation

Braak staging and atrophy data were downloaded from the supplemental data file of [22]. We then filtered the data for mRNAs that had been reported to exhibit significant correlation to either Braak staging or atrophy data in the pre-frontal cortex. Expected values were calculated by determining the total number of *proteins tested* that were reported to have significant correlations. Observed values were calculated by determining the total number of proteins *within our MMSE correlation hit list* that were reported to have significant correlations. Significance testing was performed using the chi^2^-test.

### Single Nucleotide Polymorphism (SNP) analysis

*Datasets and SNP association testing.* Summarized information from tests of genetic association of AD with SNPs located in the candidate gene regions was culled from a recent large genome-wide association study (GWAS) conducted by the Alzheimer Disease Genetics Consortium (ADGC) [23]. Naj et al. computed results for SNPs throughout the genome in their discovery sample composed of 8,309 AD cases and 7,366 cognitively normal elders from ten independent Caucasian data sets. Details of the procedures for quality control, genotype imputation, and population substructure adjustment are published elsewhere [23]. Genotyped and imputed SNPs were tested for association with AD in each dataset separately using a logistic generalized linear model (GLM) in case–control datasets and a logistic generalized estimating equation (GEE) in family-based datasets, controlling for intra-study population substructure. Genotyped SNPs were coded as 0, 1, or 2 according to the number of minor alleles under the additive genetic model. For imputed SNPs, a quantitative estimate between 0 and 2 for the dose of the minor allele were used to incorporate the uncertainty of the imputation estimates. All analyses were performed using the GEE [80] and GWAF [81] programs in the R statistical software package. SNP association results obtained from individual datasets were combined by meta-analysis using the inverse variance method implemented in the software package METAL [82] (http://www.sph.umich.edu/csg/abecasis/Metal/index.html).

### Gene-based multiple testing corrections

We corrected for testing multiple SNPs in a gene after accounting for correlation between SNP genotypes due to linkage disequilibrium. Each gene tested was treated as an independent hypothesis and the effective number of tests per gene was obtained by a previously described method [83]. The Versatile Gene-based Association Study (VEGAS) approach [84] was used to summarize the strength of association of a gene with AD based on the number of SNPs tested in the gene and size of the gene. This method computes a gene-based test statistic based on the SNP *p*-values within the gene, and then uses simulation to calculate an empirical gene-based *p*-value. The distribution of empirical *p*-values was then plotted and tested against an expected distribution of *p*-values using the Kolmogorov-Smirnov test.

### mRNA expression analysis

The dataset comprises gene expression data from brain tissues that were posthumously collected from more than 600 individuals with AD diagnosis, HD diagnosis, or with normal non-demented brains. We used a subset of dorsolateral prefrontal cortex (PFC, Brodmann area 9) samples from 181 AD case and 125 controls. Only neuropathologically confirmed AD subjects with Braak stage > III were included in this profiling experiment; Braak stage and atrophy were assessed by pathologists at McLean Hospital (Belmont, MA). The samples were flash frozen in liquid nitrogen vapor with an average postmortem interval (PMI) of about 18 h.

A total of 1 μg of mRNA extracted from each tissue sample was amplified to fluorescently labeled cRNA, and profiled by the Rosetta Gene Expression Laboratory in two phases using the Rosetta/Merck 44 k 1.1 microarray (GPL4372) (Agilent Technologies, Santa Clara, CA). The average RNA integrity number of 6.81 was sufficiently high for the microarray experiment monitoring 40,638 transcripts representing more than 31,000 unique genes. The expression levels were processed and normalized to the average of all samples in the batch from the same region using Rosetta Resolver (Rosetta Biosoftware, Seattle, WA).

All microarray data generated in this study are available through the National Brain Databank at the Harvard Brain Tissue Resource Center (http://www.brainbank.mclean.org/). This microarray dataset is MIAME compliant. The raw and final processed data for each hybridization are available upon request. The essential sample annotation including experimental factors and their values (e.g., gender, age, PMI, pH) is available and summarized in [24].

The differential gene expression was assessed using the standard *t*-test. The distribution of *p*-values was then plotted and tested against an expected distribution of *p*-values using the Kolmogorov-Smirnov test.

### Western blot

Active GDF3 levels were determined in fresh tissue samples not part of the plasma screen (Additional file 1: Table S3). Hippocampal samples were a random subset picked blindly from the same donors as the cortical samples. All tissues or cells were lysed in RIPA buffer and total protein concentrations were determined with a BCA Protein Assay Kit (Thermo Scientific, Waltham, MA). 10–20 μg of total protein was loaded for each sample into pre-cast 4–12 % bis-tris gels and run with MOPS buffer (Invitrogen, Carlsbad, CA). Gels were transferred onto PVDF membranes (Millipore, Billerica, MA). Antigen specific primary antibodies were incubated overnight at 4 °C and detected with species-specific horseradish-peroxidase labeled secondary antibodies. An ECL Western Blotting Detection kit (GE Healthcare, Cleveland, OH) was used to obtain a chemiluminescence signal, which was detected using Amersham Hyperfilm ECL (GE Healthcare). Band quantification was performed using ImageJ software (version 1.46; NIH, Bethesda, MD). Bands of interest were normalized to actin or neuron specific enolase for a loading control. For active GDF3 we used anti-GDF3 antibodies from Novus Biologicals (Littleton, CO; NBP1-96508).

### Cell culture assays

Human NTERA cells expressing eGFP under the DCX promoter were maintained in DMEM media containing 10 % FBS. To induce differentiation, cells were plated in 96 well plates. One day after seeding, 10 μM of retinoic acid and designated concentrations of recombinant carrier-free human GDF3 (R&D Systems, Minneapolis, MN; at 0, 10, 50, or 150 ng/mL) were added to the culture media of each corresponding NTERA treatment well. Cells were maintained under these conditions for 2 weeks, during which media was replaced every 3 days. Cells were then cultured for an additional 2 weeks with continued GDF3 treatment, in the absence of retinoic acid.

### Cellavista

Adult neurosphere number, eGFP expression (relative to cell confluence), and number of proliferating NPCs were quantified after GDF3 treatment using an Innovatis Cellavista Imager (Dynamic Devices, Wilmington, DE). To quantify NPC proliferation, 10x images were collected by Cellavista and BrdU+ nuclei were detected and quantified by Cellavista software using the cell nuclei count function.

### Adult hippocampal NPC isolation

Hippocampal NPCs were isolated from 6 week old male and female mice [85]. NPCs were maintained on poly-D-lysine (Sigma, St. Lous, MO) and laminin (Invitrogen) coated 10 cm plastic plates in neurobasal A media (Invitrogen) with 1× B27 supplement without vitamin A (Invitrogen) and 1× GlutaMAX-I supplement (Invitrogen) and 20 ng/ml each of recombinant human FGF-basic (Peprotech, Rocky Hill, NJ) and recombinant human EGF (Peprotech) at 37 °C and 5 % CO2. All experiments used NPCs below passage 20 and were repeated at least once with male NPCs and once with female NPCs.

### GDF3 treatment of proliferating NPCs

5000 cells were plated per well in a 96 well poly-D-lysine/laminin-coated plate with 0, 0.01, 0.1, 1, 10 or 100 ng/ml recombinant mouse GDF3 (R&D Systems) in normal growth media. Cells were allowed to grow for 4 days, with a ½ media change on day 2 (which replaced full growth factors [85] and ½ of GDF3 treatment). After 4 days in treatment, 20 μM bromodeoxyuridine (BrdU, Sigma) in sterile PBS was added to all wells and cells were fixed with 4 % paraformaldehyde 2 h later for 10 min.

### Immunocytochemistry

Fixed cells were rinsed with 0.1 M phosphate buffered saline (PBS) 3 times then blocked with 10 % normal donkey serum (NDS, Jackson ImmunoResearch, West Grove, PA) and 0.3 % Triton-X 100 (Sigma) in PBS for 30 min. Cells were incubated overnight in primary antibody, rat anti-BrdU (1:500, AbD Serotech, Raleigh NC) in 10 % NDS in PBS at 4 °C. Cells were then rinsed and incubated in secondary antibody, Alexa488 anti-rat (1:200, Invitrogen) in 10 % NDS in PBS. After rinsing, total BrdU+ cells were imaged and quantified using an automated Cellavista microscope system (Hoffman-La Roche, Basel, Switzerland).

### NPC differentiation

Murine NPCs were differentiated for 8 days [85]. Briefly, cells were plated at 200,000 cells/well in a poly-D-lysine/laminin-coated plate in either full growth factor media (20 ng/ml of EGF and FGF2; proliferative conditions) or in media with only 5 ng/ml FGF2. After 2 days, the proliferative wells and the 2d differentiation wells were harvested while the 4d and 8d wells received a complete media change to media with no growth factors added. At day 4, the 4d wells were harvested and the 8d wells received a ½ media change with no growth factors added. A ½ media change was repeated on day 6 and the remaining wells were harvested on day 8.

### RNA harvesting and conversion to cDNA

Cells were removed from the plate using Accutase Cell Dissociation Reagent (Invitrogen) then centrifuged at 400 g for 5 min. The cell pellet was stored at −80 °C until later RNA extraction. RNA extraction was performed using the RNeasy Mini Kit (Qiagen, Venlo, Netherlands) as per manufacturer instructions. The resulting RNA was quantified using a nanodrop spectrophotometer and RNA purity was confirmed using A260/A280 ratios. 500 ng of RNA was treated with DNase I as per manufacturer instructions (Invitrogen) to eliminate any genomic DNA contamination and then converted to cDNA using SuperScript III first-strand synthesis system (Invitrogen) as per manufacturer instructions. cDNA was diluted 1:5 in water.

### Real-time quantitative PCR

2 μl of cDNA was quantified in duplicate for each sample using LightCycler 480 SYBR Green I (Roche) on a LightCycler 480 II as per manufacturer instructions. Cycling conditions were: 15 min at 95 °C, 45 cycles of [15 s at 94 °C, 25 s at 58 °C, 20 s at 72 °C]. Melt curve cycles immediately followed and were: 5 s at 95 °C, 1 min at 65 °C and then gradual temperature rise to 97 °C at a rate of 0.11 °C/s followed by 30s at 40 °C. GDF3 levels were normalized to MAPK3 [86] as a reference gene because MAPK3 has been shown not to change with differentiation in contrast to many other standard housekeeping genes such as actin, which change dramatically during the differentiation process [87]. Melt curve analysis was performed to verify primer specificity and all primers were tested in a dilution series before use. Data is displayed as fold change above proliferative condition mRNA levels using 2^(ΔΔCt) values.

Primer sequences were obtained from the MIT/Harvard PrimerBank.

GDF3 fwd: 5’ ATGCAGCCTTATCAACGGCTT

GDF3 rev: 5’ AGGCGCTTTCTCTAATCCCAG

GDF3 PrimerBankID: 6679979a1

MAPK3 fwd: 5’ TCCGCCATGAGAATGTTATAGGC

MAPK3 rev: 5’ GGTGGTGTTGATAAGCAGATTGG

MAPK3 PrimerBankID: 21489933a1

## Additional files

Additional file 1: Patient and antibody information, raw and processed data. (XLSX 6303 kb) Additional file 2: Supplemental demographic information **Tables S1-S3** and additional **Figures S1-S7**. (PDF 2603 kb)

## Abbreviations

AD: Alzheimer’s disease
BMP: bone morphogenetic protein
BrdU: bromodeoxyuridine
Dcx: doublecortin
FTLD: frontotemporal lobar degeneration
GDF: growth-differentiation factor
GO: gene ontology
MMSE: mini-mental state examination
NPC: neural progenitor cells
SNP: single nucleotide polymorphism
svPPA: semantic-variant primary progressive aphasia
