# Erratum to: Network-driven plasma proteomics expose molecular changes in the Alzheimer’s brain

**DOI:** 10.1186/s13024-016-0105-4

**Published:** 2016-05-23

**Authors:** Philipp A. Jaeger, Kurt M. Lucin, Markus Britschgi, Badri Vardarajan, Ruo-Pan Huang, Elizabeth D. Kirby, Rachelle Abbey, Bradley F. Boeve, Adam L. Boxer, Lindsay A. Farrer, NiCole Finch, Neill R. Graff-Radford, Elizabeth Head, Matan Hofree, Ruochun Huang, Hudson Johns, Anna Karydas, David S. Knopman, Andrey Loboda, Eliezer Masliah, Ramya Narasimhan, Ronald C. Petersen, Alexei Podtelezhnikov, Suraj Pradhan, Rosa Rademakers, Chung-Huan Sun, Steven G. Younkin, Bruce L. Miller, Trey Ideker, Tony Wyss-Coray

**Affiliations:** Department of Neurology and Neurological Sciences, Stanford University School of Medicine, Stanford, CA USA; Institute of Chemistry and Biochemistry, Free University, Berlin, Berlin Germany; Departments of Bioengineering and Medicine, University of California, La Jolla, San Diego, CA USA; Department of Medicine (Biomedical Genetics), Boston University Schools of Medicine, Boston, MA USA; RayBiotech, Guangzhou, China; RayBiotech, Norcrosse, GA USA; Department of Neurology, Mayo Clinic, Rochester, MN USA; Department of Neurology, University of California San Francisco, San Francisco, CA USA; Departments of Neurology, Ophthalmology, Genetics and Genomics, Epidemiology, and Biostatistics, Boston University Schools of Medicine and Public Health, Boston, MA USA; Department of Neuroscience, Mayo Clinic, Jacksonville, FL USA; Department of Neurology, Mayo Clinic, Jacksonville, FL USA; Departments of Pharmacology and Nutritional Sciences and Sanders-Brown Center on Aging, University of Kentucky, Lexington, KY USA; Department of Computer Science and Engineering, University of California, La Jolla, San Diego, CA USA; Genetics and Pharmacogenomics, Merck Research Laboratories, West Point, PA USA; Department of Pathology, University of California, La Jolla, San Diego, CA USA; Center for Tissue Regeneration, Repair and Restoration, VA Palo Alto Health Care System, Palo Alto, CA USA; Present address: Biology Department, Eastern Connecticut State University, Willimantic, CT USA; Present address: Roche Pharma Research and Early Development, NORD DTA, Roche Innovation, Center Basel, Basel, Switzerland

Unfortunately, after publication of this article [1], it was noticed that the name of Matan Hofree was misspelled. The corrected name can be seen above and the original article has been updated to reflect this change.
