# Supplementary material for: Network-driven plasma proteomics expose molecular changes in the Alzheimer’s brain
Source: Mol Neurodegener. 2016 Apr 26;11:31. doi: 10.1186/s13024-016-0095-2 (PMC4845325; doi:10.1186/s13024-016-0095-2)
Supplement: Additional file 2: — Supplemental demographic information Tables S1-S3 and additional Figures S1-S7. (PDF 2603 kb) [file 13024_2016_95_MOESM2_ESM.pdf]

Supporting Information for

Jaeger et al., *Network-driven plasma proteomics expose molecular changes in the Alzheimer's brain*

Content:

- **Tables S1-S3**
- **Figures S1-S7**
- **Supplemental Data File description**
- **Funding Information**
- **Supplemental References**

Table S1: Subject demographics of AD plasma donors

**Condition**	**Control**		**AD**	
**Gender**	Male	Female	Male	Female
**N Subjects**	28 (53%)	24 (47%)	26 (55%)	21 (45%)
	52		47[a]	
**Mean age (range)**	68.9 ± 9.4 (45.6-86)		69.2 ± 9.8 [n.s.] (51.3-85)	
**Mean MMSE**	29.7 ± 0.6 (28-30)[b]		18.3 ± 6.9 *** (4-29)[b]	
**ApoE status**	2/3: 2 (4%)<br>2/4: - (0%)<br>3/3: 31(60%)<br>3/4: 15 (29%)<br>4/4: 1 (2%)<br>NA: 3 (6%)		2/3: 2 (4%)<br>2/4: 3 (6%)<br>3/3: 8 (17%)<br>3/4: 21 (45%)<br>4/4: 5 (11%)<br>NA: 8 (17%)	
**Sample origin**	UCSF: 21 (40%)<br>Mayo: 31 (60%)		UCSF: 20 (43%)<br>Mayo: 27 (57%)	

Plasma samples were collected at two centers (The Mayo Clinic, Rochester, MN and Jacksonville, FL; University of California San Francisco, San Francisco, CA). Data are 'mean ± standard deviation (range)' or 'number (%)', respectively; n.s., not significant; *** $p < 0.0001$ (Student t-test). [a]Post-mortem confirmation was available for 27 of the AD subjects. [b]MMSE scores were available for 26 Control and 44 AD subjects.

Table S2: Subject demographics of svPPA plasma donors

**Condition**	**Control**		**svPPA**	
**Gender**	Male	Female	Male	Female
**N Subjects**	41 (49%)	42 (51%)	45 (49%)	47 (51%)
	83		92[a]	
**Mean age in years (range)**	66.7 ± 9.1 (43-86)		67.4 ± 9.1 [n.s.] (41-86)	
**Mean MMSE**	29.6 ± 0.6 (28-30)[b]		21.1 ± 8.5 *** (0-30)[b]	
**Sample origin**	UCSF: 31 (37%) Mayo: 52 (63%)		UCSF: 33 (36%) Mayo: 59 (64%)	

Plasma samples were collected at two centers (The Mayo Clinic, Rochester, MN and Jacksonville, FL; University of California San Francisco, San Francisco, CA); Data are 'mean ± standard deviation (range)' or 'number (%)', respectively; n.s., not significant; *** $p=2.2x10^{-10}$ (Student t-test). [a]Post-mortem confirmation was available for 35 of the svPPA subjects. [b] MMSE scores were available for 39 Control and 58 svPPA subjects.

Table S3: Subject demographics of the confirmation brain tissue samples

Condition	Control for AD		AD		Control for svPPA		svPPA	
**Gender**	Male	Female	Male	Female	Male	Female	Male	Female
**N subjects**	70%	30%	75%	25%	60%	40%	80%	20%
	18		16		5		5	
**Mean age in years (range)**	76 ± 7.6 (68-91)		77.6 ± 7.7 (65-89) n.s.		71.8 ± 12.4 (50-88)		70.8 ± 5.9 (60-78) n.s.	
**Mean MMSE**	29.4 ± 0.5 (29-30)		5.4 ± 5.5 (0-14) ***		n.a.		n.a.	
**Sample origin**	UCSD: 100%		UCSD: 100%		UCI: 100%		UCI: 100%	

Cortical and hippocampal tissue samples were collected at two centers (University of California San Diego, La Jolla, CA; University of California Irvine, Irvine, CA); Data are 'mean ± standard deviation (range)' or 'number (%)', respectively; n.s., not significant; n.a., not available; *** p<0.0001 (Student t-test).

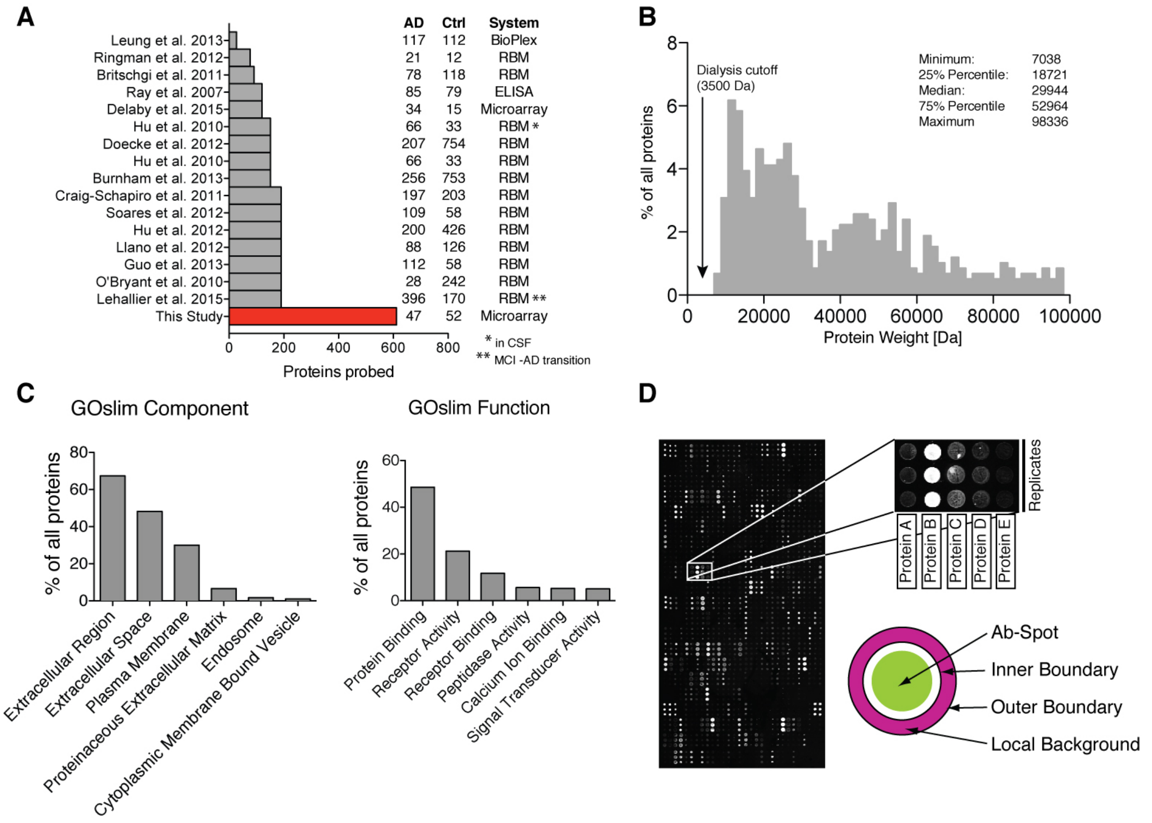

Figure S1: Study parameters. (A) Comparison of this study to other recent multiplex AD studies in human blood/CSF samples. Number next to bar indicates sample size (only antibody-based studies with samples>20 and analytes>30 are shown). [1-15] **(B)** Protein size distribution (based on calculated molecular weight). **(C)** Gene ontology distribution. The proteins used in this screen were classified by their gene ontology annotation, demonstrating the secreted or secretable nature of the proteins measured (proteins can belong to more than one category). **(D)** Example of a scanned array. Antibodies were printed at least in triplicates. Spot intensity was measured in a region confined by the inner boundary. The area between the inner and outer boundaries served as local background for normalization.

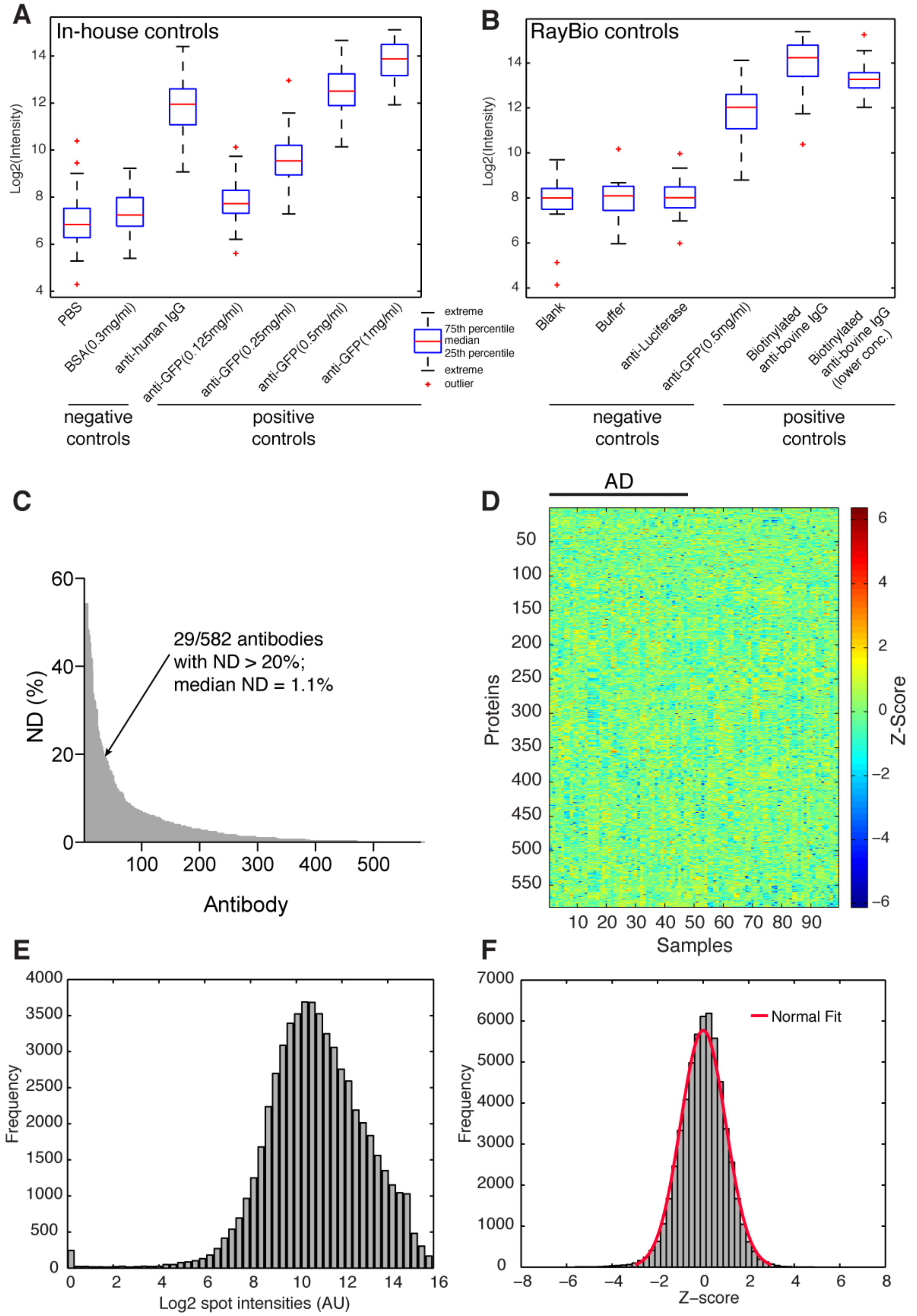

Figure S2: Controls and data transformation. (A) Microarray controls of the in-house array (raw intensity). Negative controls of buffer alone (PBS) or protein

alone (BSA) were printed to assess non-specific binding. Positive controls consisted of anti-human IgG antibody and a concentration gradient of anti-GFP antibody against spiked recombinant protein. **(B)** Microarray controls of the RayBio array (raw intensity). Negative controls of pin-touch (blank), buffer alone (PBS), or antibody alone (anti-luciferase) were printed to asses non-specific binding. Positive controls consisted of anti-GFP and biotinylated anti-bovine IgG (binds the detection reagent). **(C)** Distribution of spots below the detection level (ND = non-detectable) per antibody analyzed. For downstream analysis, these spots were replaced with the half-detection limit. **(D)** Heat map of the data after normalization steps. **(E)** Distribution of raw intensity values. **(F)** Distribution of normalized data. *Panels (E) and (F) were adapted from* [16] *with the permission of the publisher.*

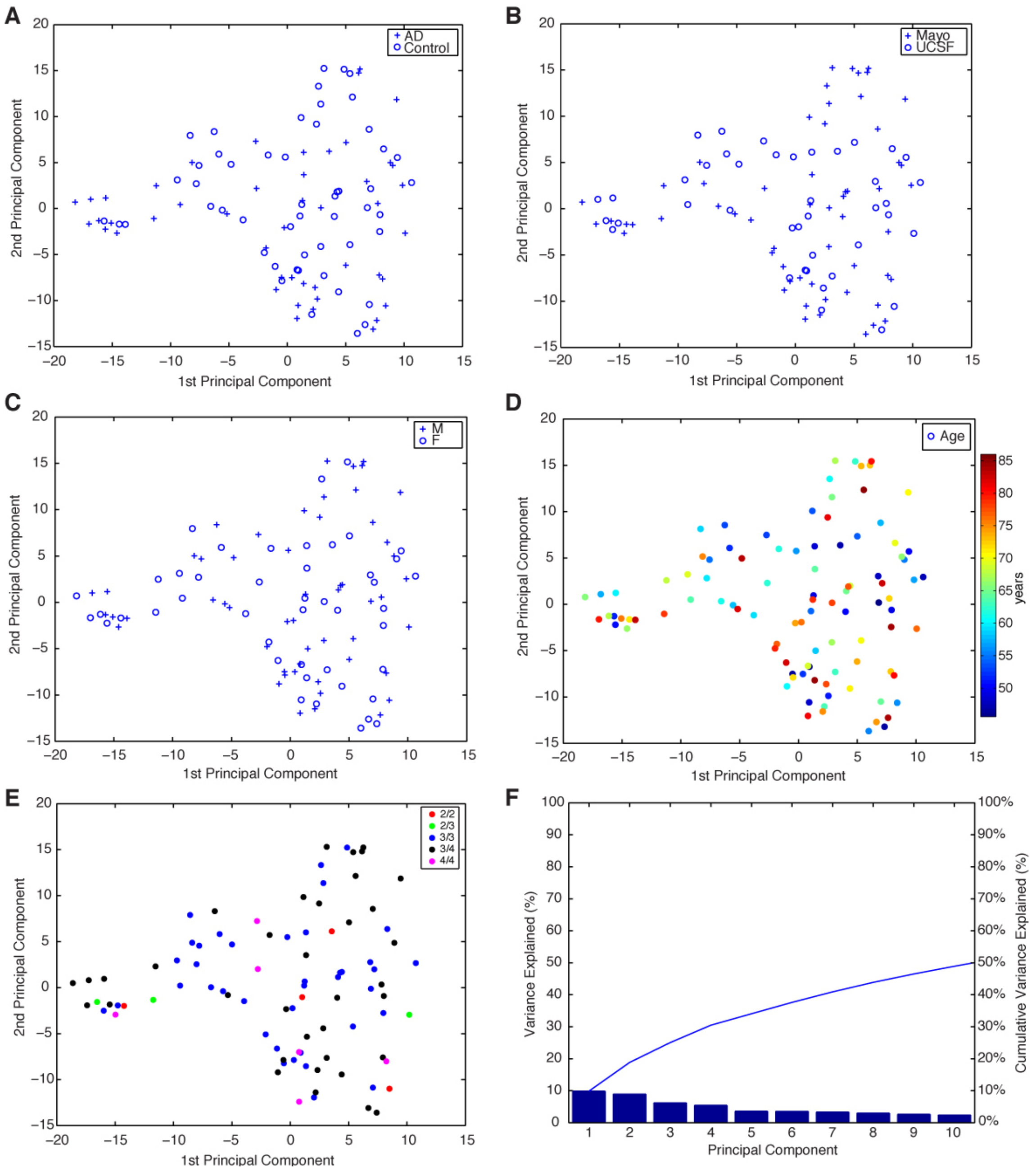

Figure S3: Principle Component Analysis of the Z-scored data. We performed PCA to assess the influence of potential confounding factors. PCA revealed no obvious clustering when applied to the normalized 582 antibody input data relative to disease status **(A)**, clinical center **(B)**, sex **(C)**, age **(D)**, or ApoE genotype. **(E)** Variance explained by the first ten principal components.

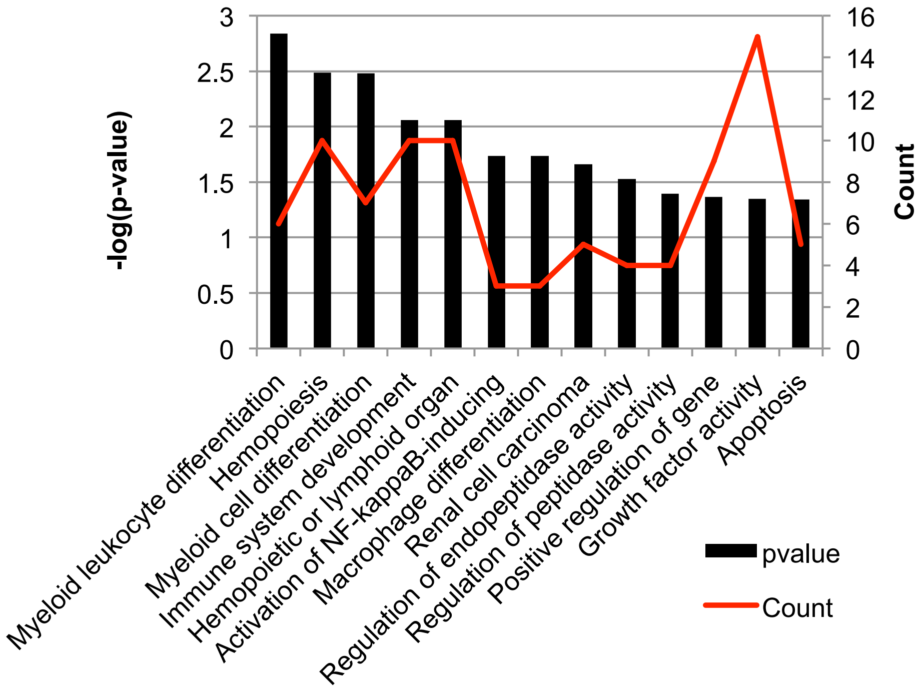

Figure S4: Ontology Enrichment of the top proteins. The top proteins (FDR<0.05, corresponding to p_{corr}<0.015) were annotated for functional enrichment using GeneOntology Biological Porcess, Molecular Function, and KEGG Pathway. For details see **Supplemental Data File**.

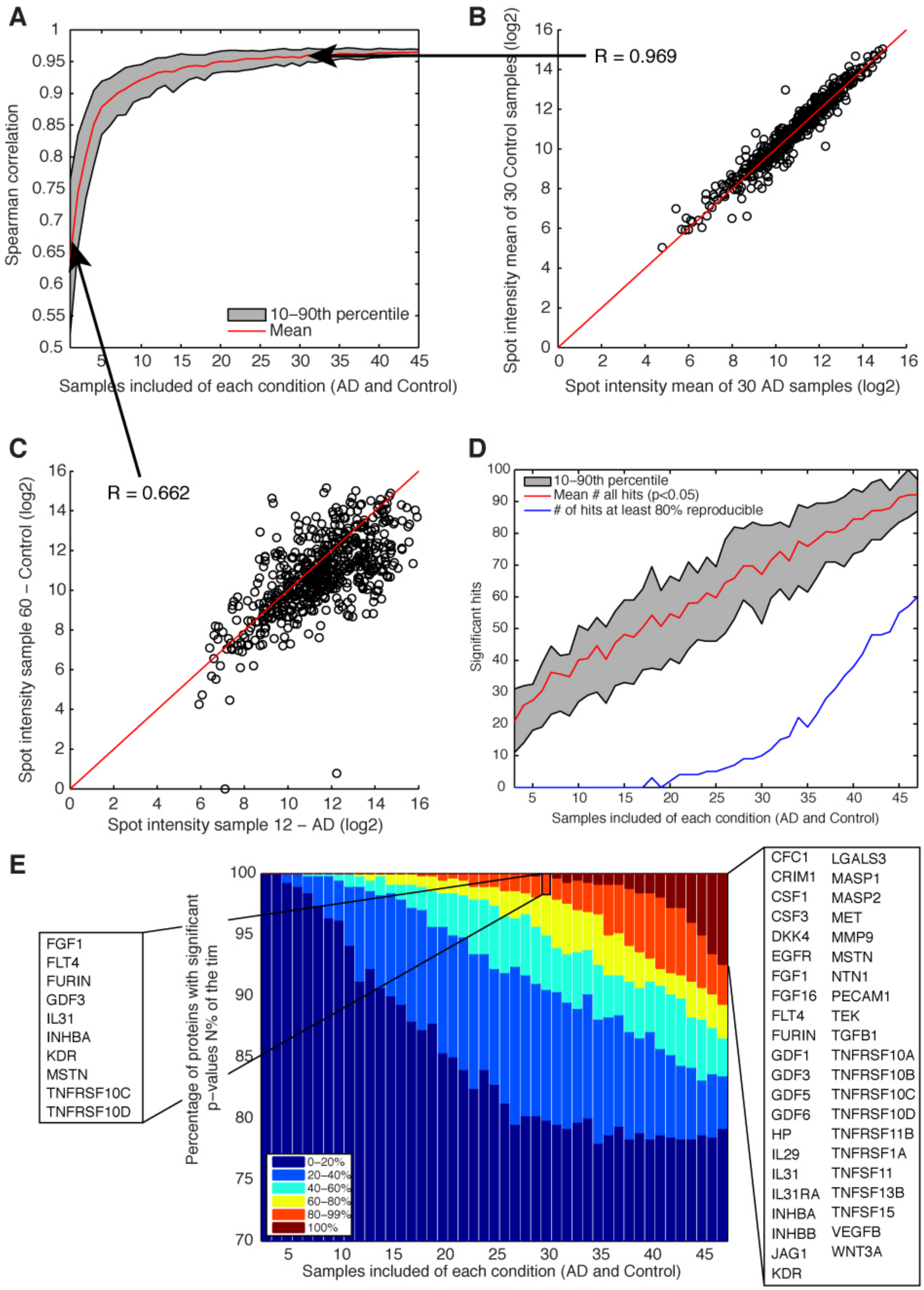

Figure S5: Data consistency and hit quality (leave-N-out). To assess the consistency of the array signals we assumed that most tested antibody-protein pairs do not change between AD and Control samples and thus array-to-array correlation should be significant. **(A)** We sampled AD and Control arrays

randomly 100 times and compared spot intensity correlations between individual arrays (samples = 1) or averaged spot intensities of several arrays (samples > 1). While array-to-array correlation is already very good, a minimum of 5-10 samples per cohort is needed to reduce technical noise. **(B) - (C)** Examples of correlation between sets of 30 averaged arrays per condition (B) or one array each (C). ***Note:*** *Adding more samples will not increases cohort correlation much further, but can be very useful downstream for significance testing of potential hits.* **(D)** Effect of sample size on number of significant hits: Increasing the sample size continuously increases the number of significant hits (red line, mean of 100 random samplings). However, most of these 'hits' are not stable (i.e. are only observed a few times in the 100 samplings). True positive hits (hits in >80% of the samplings) are found at cohort sizes greater than approximately 20 samples each (blue line). **(E)** Distribution of hit quality in response to sample size: At small sample sizes, all hits are unstable (i.e. occur only infrequently or randomly 0-60% of the samplings; shades of blue). At cohort sizes of 20 and above, true positive hits begin to be identifiable (i.e. occur in most samplings 80-100%; red and orange). Example high confidence hits of cohort sizes 30 AD and 30 Controls (left box) as well as 47 AD and 47 Controls (right box) are shown. ***Note:*** *If one extrapolates the 100% occurrence border (red) and the 0-20% occurrence border (dark blue) than it can be estimated that at about 65 samples in each cohort the population hits would be binary (i.e. red and dark blue meet) with about 15% (87 proteins) true-positive hits and 85% non-hits. Given the power of our study (47 AD / 52 Controls), we can expect to have recovered at least ~43/87 of the true positives (100% occurrence) and an additional ~20 very likely true positives (80-99% occurrence). Overall our study has likely recovered ~75% (63/87) of the true positive hits for this screening format.*

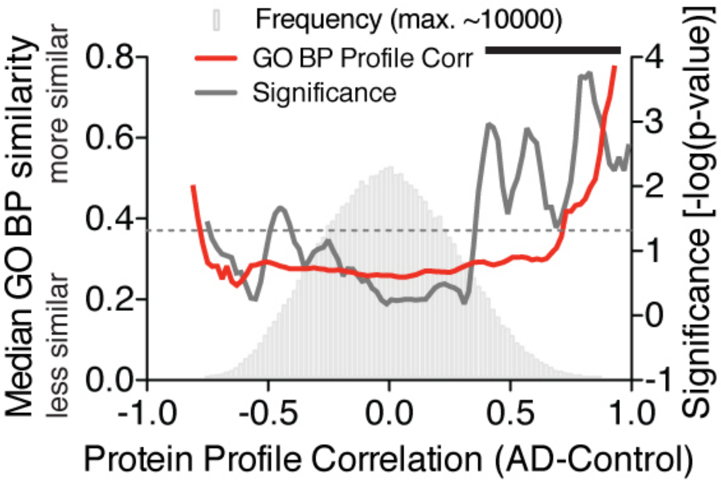

Figure S6: Biological content of differential co-expression profiles. We calculated pairwise semantic similarity score of the protein pairs from ~0.1 (very different) to ~0.9 (very similar) in the "biological process" gene ontology as a measure for distance in the ontology tree and shared membership in biological processes (used as a silver standard for benchmarking). To benchmark the concept of "protein profile correlation" we assessed whether protein pairs with high differential co-expression profile correlation are enriched for protein pairs with high GO BP profile similarity (significance based on bootstrapping with 10,000 permutations; grey histogram of underlying protein pair correlation distribution is not mapped to any y-axis; black bar indicates area of significant enrichment). This indicates that biologically meaningful data can be gathered from these profiles to identify related protein groups that could drive the observed changes in protein correlation trends.

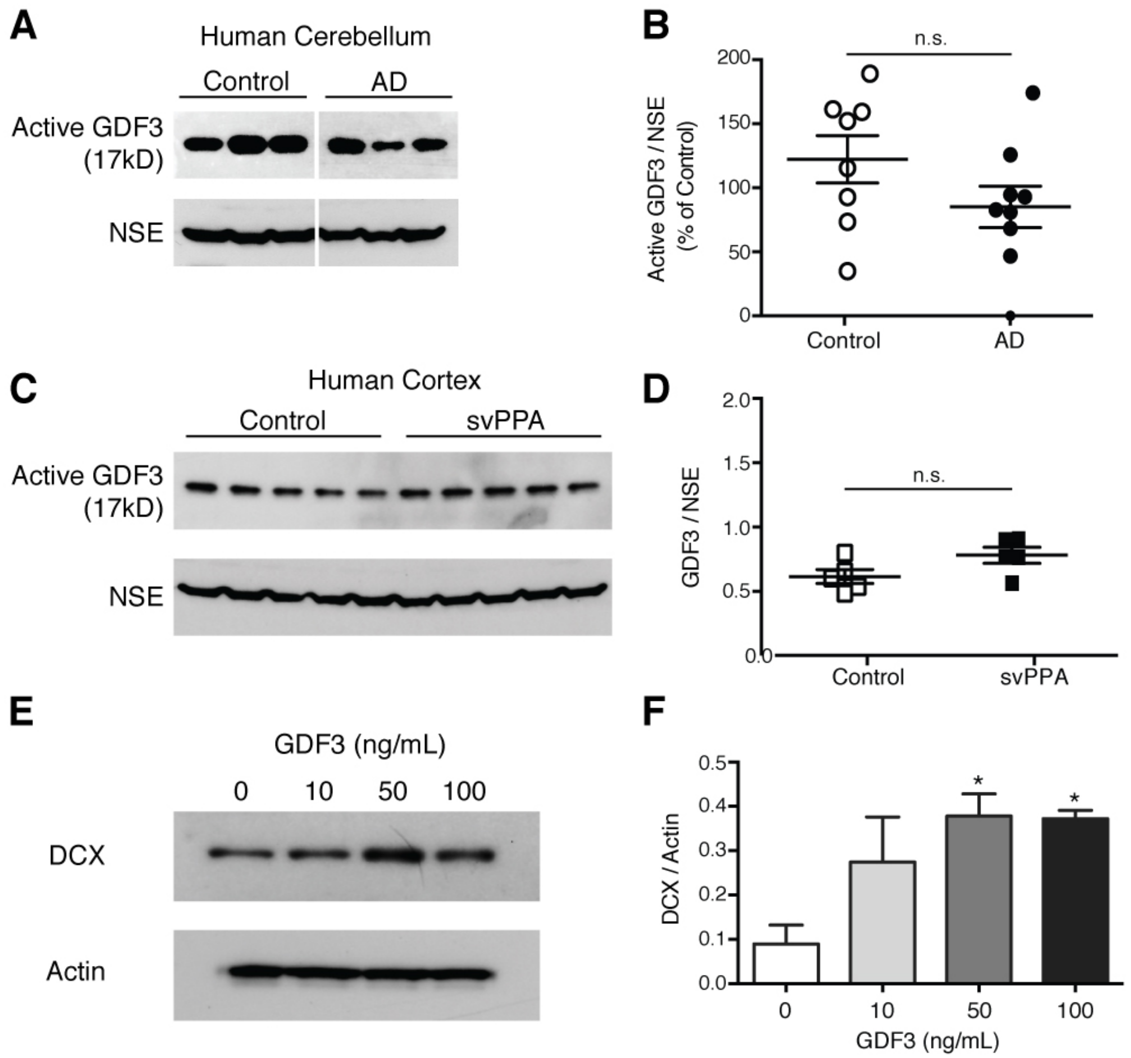

Figure S7: Protein quantification and effect of GDF3 in additional samples. To assess whether reduced GDF3 levels are unique to affected AD brain regions, GDF3 was also quantified in non-affected cerebellum tissue from human AD patients and cortex from human svPPA patients. **(A) & (B)** Cerebellar tissues from human AD and non-demented controls (N=8-9 per group). **(C) & (D)** Cortical tissues from human svPPA and non-demented controls (mid-frontal gray matter) were lysed and the detergent soluble protein fraction was probed by western blot (N=5 per group). In (A) to (F) levels of active GDF3 were quantified relative to neuron-specific enolase (NSE). **(E) & (F)** To confirm DCX-GFP fluorescence expression, human-derived NTERA cells were treated with recombinant human GDF3 or control for 30 days in vitro. Cells were then lysed and the detergent soluble protein fraction was probed by western blot. Levels of DCX were quantified relative to actin (N=3 per group). All results were compared by an unpaired Student's t-test. Values are mean ± s.e.m.. * $p<0.05$, ** $p<0.01$, n.s., not significant ($p>0.05$).

SUPPLEMENTAL DATA FILE

The Supplemental Data File contains detailed information on the patient samples used for this study, the un-normalized microarray data, the normalized microarray data, a detailed list of the statistical results of the differential protein level and the MMSE correlation analyses, the results from the mRNA and SNP analyses, as well as background and quality information on the 582 antibodies analyzed.

SUPPLEMENTAL REFERENCES

1. Leung R, Proitsi P, Simmons A, Lunnon K, Güntert A, Kronenberg D, Pritchard M, Tsolaki M, Mecocci P, Kloszewska I: **Inflammatory proteins in plasma are associated with severity of Alzheimer's disease**. *PLoS ONE* 2013, **8**:e64971.

2. Ringman JM, Elashoff D, Geschwind DH, Welsh BT, Gylys KH, Lee C, Cummings JL, Cole GM: **Plasma signaling proteins in persons at genetic risk for Alzheimer disease: influence of APOE genotype.** *Arch Neurol* 2012, **69**:757–764.

3. Britschgi M, Rufibach K, Huang SLB, Clark CM, Kaye JA, Li G, Peskind ER, Quinn JF, Galasko DR, Wyss-Coray T: **Modeling of pathological traits in Alzheimer's disease based on systemic extracellular signaling proteome**. *Mol Cell Proteomics* 2011, **10**:M111. 008862.

4. Ray S, Britschgi M, Herbert C, Takeda-Uchimura Y, Boxer A, Blennow K, Friedman LF, Galasko DR, Jutel M, Karydas A, Kaye JA, Leszek J, Miller BL, Minthon L, Quinn JF, Rabinovici GD, Robinson WH, Sabbagh MN, So YT, Sparks DL, Tabaton M, Tinklenberg J, Yesavage JA, Tibshirani R, Wyss-Coray T: **Classification and prediction of clinical Alzheimer's diagnosis based on plasma signaling proteins.** *Nat Med* 2007, **13**:1359–1362.

5. Delaby C, Gabelle A, Blum D, Schraen-Maschke S, Moulinier A, Boulanghien J, Séverac D, Buée L, Rème T, Lehmann S: **Central Nervous System and Peripheral Inflammatory Processes in Alzheimer's Disease: Biomarker Profiling Approach**. *Front Neurol* 2015, **6**:103.

6. Hu WT, Chen-Plotkin A, Arnold SE, Grossman M, Clark CM, Shaw LM, Pickering E, Kuhn M, Chen Y, Mccluskey L, Elman L, Karlawish J, Hurtig HI, Siderowf A, Lee VM-Y, Soares H, Trojanowski JQ: **Novel CSF biomarkers for Alzheimer's disease and mild cognitive impairment**. *Acta Neuropathol* 2010, **119**:669–678.

7. Doecke JD, Laws SM, Faux NG, Wilson W, Burnham SC, Lam C-P, Mondal A, Bedo J, Bush AI, Brown B, De Ruyck K, Ellis KA, Fowler C, Gupta VB, Head R,

Macaulay SL, Pertile K, Rowe CC, Rembach A, Rodrigues M, Rumble R, Szoeke C, Taddei K, Taddei T, Trounson B, Ames D, Masters CL, Martins RN, Alzheimer's Disease Neuroimaging Initiative, Australian Imaging Biomarker and Lifestyle Research Group: **Blood-based protein biomarkers for diagnosis of Alzheimer disease.** *Arch Neurol* 2012, **69**:1318–1325.

8. Hu WT, Holtzman DM, Fagan AM, Shaw LM, Perrin R, Arnold SE, Grossman M, Xiong C, Craig-Schapiro R, Clark CM, Pickering E, Kuhn M, Chen Y, Van Deerlin VM, Mccluskey L, Elman L, Karlawish J, Chen-Plotkin A, Hurtig HI, Siderowf A, Swenson F, Lee VM-Y, Morris JC, Trojanowski JQ, Soares H, Initi ADN: **Plasma multianalyte profiling in mild cognitive impairment and Alzheimer disease**. *Neurology* 2012, **79**:897–905.

9. Burnham SC, Faux NG, Wilson W, Laws SM, Ames D, Bedo J, Bush AI, Doecke JD, Ellis KA, Head R, Jones G, Kiiveri H, Martins RN, Rembach A, Rowe CC, Salvado O, Macaulay SL, Masters CL, Villemagne VL, Initiative ARSDN, Australian Imaging BALSRG: **A blood-based predictor for neocortical Ab burden in Alzheimer's disease: results from the AIBL study**. *Mol Psychiatry* 2013, **19**:519–526.

10. Craig-Schapiro R, Kuhn M, Xiong C, Pickering EH, Liu J, Misko TP, Perrin RJ, Bales KR, Soares H, Fagan AM: **Multiplexed immunoassay panel identifies novel CSF biomarkers for Alzheimer's disease diagnosis and prognosis**. *PLoS ONE* 2011, **6**:e18850.

11. Soares HD, Potter WZ, Pickering E, Kuhn M, Immermann FW, Shera DM, Ferm M, Dean RA, Simon AJ, Swenson F, Siuciak JA, Kaplow J, Thambisetty M, Zagouras P, Koroshetz WJ, Wan HI, Trojanowski JQ, Shaw LM, Biomarkers Consortium Alzheimer's Disease Plasma Proteomics Project: **Plasma biomarkers associated with the apolipoprotein E genotype and Alzheimer disease.** *Arch Neurol* 2012, **69**:1310–1317.

12. Llano DA, Devanarayan V, Simon AJ, ADNI: **Evaluation of Plasma Proteomic Data for Alzheimer Disease State Classification and for the Prediction of Progression From Mild Cognitive Impairment to Alzheimer Disease**. *Alzheimer Dis Assoc Disord* 2013, **27**:233–243.

13. Guo L-H, Alexopoulos P, Wagenpfeil S, Kurz A, Perneczky R, Neuroimaging AD: **Plasma Proteomics for the Identification of Alzheimer Disease**. *Alzheimer Dis Assoc Disord* 2013, **27**:337–342.

14. O'Bryant SE, Xiao G, Barber R, Reisch J, Doody R, Fairchild T, Adams P, Waring S, Diaz-Arrastia R, for the Texas Alzheimer's Research Consortium: **A Serum Protein-Based Algorithm for the Detection of Alzheimer Disease**. *Arch Neurol* 2010, **67**:1077–1081.

15. Lehallier B, Essioux L, Gayan J, Alexandridis R, Nikolcheva T, Wyss-Coray T, Britschgi M, for the Alzheimer's Disease Neuroimaging Initiative: **Combined Plasma and Cerebrospinal Fluid Signature for the Prediction of Midterm Progression From Mild Cognitive Impairment to Alzheimer Disease**. *JAMA Neurol* 2015:1.

16. Jaeger PA, Villeda SA, Berdnik D, Britschgi M, Wyss-Coray T: **Focused Plasma Proteomics for the Study of Brain Aging and Neurodegeneration**. In *The OMICS: Applications in Neuroscience*. Edited by Coppola G. New York: Oxford Univ. Press; 2014:183–191.
